# Inclinometer Assembly Error Calibration and Horizontal Image Correction in Photoelectric Measurement Systems

**DOI:** 10.3390/s18010248

**Published:** 2018-01-16

**Authors:** Xiaofang Kong, Qian Chen, Jiajie Wang, Guohua Gu, Pengcheng Wang, Weixian Qian, Kan Ren, Xiaotao Miao

**Affiliations:** 1 Jiangsu Key Laboratory of Spectral Imaging and Intelligent Sense, School of Electronic and Optical Engineering, Nanjing University of Science and Technology, Nanjing 210094, China; xiaofangkong@163.com (X.K.); njustwangjj@163.com (J.W.); gghnjust@mail.njust.edu.cn (G.G.); wpch08@njust.edu.cn (P.W.); developer_plus@163.com(W.Q.); k.ren@njust.edu.cn (K.R.); 2 Jilin Jiangji Special Industries Co., Ltd., Zun Yi West Road 17#, Jilin 132021, China; jian-xin0704@163.com

**Keywords:** photoelectric measurement, inclinometer assembly error calibration, plumb line, horizontal image correction, Euler algorithm

## Abstract

Inclinometer assembly error is one of the key factors affecting the measurement accuracy of photoelectric measurement systems. In order to solve the problem of the lack of complete attitude information in the measurement system, this paper proposes a new inclinometer assembly error calibration and horizontal image correction method utilizing plumb lines in the scenario. Based on the principle that the plumb line in the scenario should be a vertical line on the image plane when the camera is placed horizontally in the photoelectric system, the direction cosine matrix between the geodetic coordinate system and the inclinometer coordinate system is calculated firstly by three-dimensional coordinate transformation. Then, the homography matrix required for horizontal image correction is obtained, along with the constraint equation satisfying the inclinometer-camera system requirements. Finally, the assembly error of the inclinometer is calibrated by the optimization function. Experimental results show that the inclinometer assembly error can be calibrated only by using the inclination angle information in conjunction with plumb lines in the scenario. Perturbation simulation and practical experiments using MATLAB indicate the feasibility of the proposed method. The inclined image can be horizontally corrected by the homography matrix obtained during the calculation of the inclinometer assembly error, as well.

## 1. Introduction

The photoelectric measurement system detects and measures objects by placing a visible CCD or an infrared CCD in a fixed site. Due to terrain variations in the shooting range, a variety of inertial sensors or inclinometers are needed to assist attitude calculation [1,2,3,4,5] and image stabilization [6] for the detectors in the system. With the rapid development and wide application of these devices, the calibration of photoelectric measurement systems is becoming a hot research topic both domestically and overseas, as they are widely used in aerospace, navigation [7,8,9,10,11], automotive robots [12,13], and many other fields [14]. Low cost [3,4,5], high precision, small size, low power dissipation, high overload and high reliability are the directions in which the development of photoelectric measurement systems is focused [15,16,17,18].

Detectors in photoelectric measurement systems should have high angular resolution, which increases the measurement accuracy. During the process of calculating the camera attitude information, the assembly error of the inertial sensor or inclinometer [10] and other system interference factors should be considered and calibrated first, and then combined into the calculation to obtain the desired system precision [15,19].

On the one hand, the inclinometer is a measuring implement used for small-angle measurement [20]. It can be fixed in photoelectric measurement systems to obtain attitude changes relative to the system’s horizontal plane [21]. The real position of the object to be measured in the three-dimensional space coordinate system can be calculated by modeling the variables obtained from the inclinometer and combining them with image processing methods [10,22].

However, the inclinometer can only measure the angle between the assembly plane and the horizontal plane, and cannot acquire complete system attitude information. Thus, in order to perform assembly error calibration, inertial sensors are often needed for assistance. Nevertheless, the drawback is that the cumulative errors of the inertial sensor itself will affect the accuracy of the system’s assembly error calibration [23]. Additionally, the cost of high-precision inertial sensors or gyroscopes is very high, and they are not convenient for system integration. 

On the other hand, with regard to the field of photography [14] and image processing in computer vision, in order to meet some demands while taking photos—for example, taking panoramic [24] or distant-view pictures—the camera needs to remain a fixed degree. Additionally, when using functions based on multiple exposures or bracketed exposures for shooting, the pictures will not overlap if the levels are not the same for two exposures, creating ghosting images. Furthermore, sometimes, objects in the image are inclined due to shaking of the camera, or other factors while capturing. For example, some buildings are not perpendicular to the ground, edges of the door and wall are not perpendicular to the floor, etc. These images will not look correct if horizontal correction is not performed. Therefore, it is necessary to pay attention to the level of the camera. Through the use of an inclinometer in the photoelectric system, the image can be horizontally corrected, and the design of the picture can be optimized.

In this paper, a new method for inclinometer assembly error calibration and horizontal image correction is proposed based on plumb lines in the scenario. During the calibration, only images with plumb lines captured by the camera and inclination data from the inclinometer are needed. This paper firstly analyzes the expression of the inclinometer assembly error in photoelectric measurement systems, and then establishes the optimization function of the inclinometer assembly error according to the principle that “the plumb lines of edges of constructions in the real world should become vertical lines on the image plane after horizontal correction by the inclinometer”. Finally, the image is horizontally corrected to verify the accuracy of the calibration result, and calibration errors are discussed through simulation and practical experiments.

The structure of this paper is as follows. Section 2 provides the related work on applications of the camera and the inclinometer, as well as the contributions made by the proposed method. Section 3 explains inclinometer assembly error in photoelectric measurement systems. Section 4 introduces the proposed calibration and horizontal image correction method based on plumb lines. Section 5 shows the experimental results and analyzes the system errors. Section 6 is the conclusion.

## 2. Related Work and Motivation

Much progress has been witnessed in recent years in inertial and inclinometer calibration, and their applications in various areas. 

Merckel [25], et al. described a method for finding the pose of an object from a single image using an inclinometer attached to the camera to reduce the number of unknown parameters during the exact pose calculation, and efficiently compute the pose by using a classical iterative optimization method. 

Chang [26], et al. introduced a four-parameter mathematical computing model for the state parameters of the inclinometer, and analyzed various calculation models for the state parameters of the inclinometer.

Hirata [27], et al. presented a method for measuring the thoracic kyphosis angle in an upright standing position with a digital inclinometer and a digital camera. Results showed that the thoracic kyphosis angle of the digital inclinometer was significantly correlated with the angle derived from the digital image analysis, which suggested that a digital inclinometer is an instrument that is simple and easy to use for measuring the thoracic kyphosis angle in the clinic. 

In Ref [10], Fabio, et al. proposed a method of using the altitude of celestial bodies to determine position in unknown territory. The experimental setup was similar to that used in our paper, consisting of a camera, a digital inclinometer, neutral density filters and an adjustable platform. The method obtains the position on Earth based on the principle that “a set of circles of equal altitude can be intersected to yield viewer position”. The measurements are processed by non-linear least-squares optimization, replacing the tables used by mariners [28], and which comprises one of the biggest innovations of this method. This paper also analyzed system calibration, providing much inspiration with regard to error analysis.

Motivated by the currently available methods, a new method for inclinometer assembly error calibration and horizontal image correction is proposed based on plumb lines in the scenario. The major contributions of the method proposed in this paper can be summarized as follows.

(1) Based on the principle that “the plumb lines of edges of constructions in the real world should become vertical lines on the image plane after horizontal correction of the attitude of the camera by the inclinometer”, only the angle information of the different attitudes obtained by the inclinometer and the plumb lines in the acquired images are needed to calibrate the inclinometer assembly error matrix in the photoelectric system, which is fast and easy.
(2) The inclinometer assembly error matrix expression in photoelectric systems is analyzed in this paper, and an optimization function to achieve the optimal solution for the assembly error matrix by minimizing the Sum of Squared Residuals (SSR) is established.
(3) A captured image with an arbitrary inclination angle can be horizontally corrected after the system calibration in order to test the correctness of the calibration result.
(4) Factors affecting the accuracy of the calibration results are analyzed by means of a simulation perturbation experiment and a practical experiment, which show sufficient accuracy for the proposed method.
(5) The experimental setup is simple to implement. The calibration process is easily operated. The experimental results are stable and effective.

## 3. Inclinometer Assembly Error 

**Figure 1:**
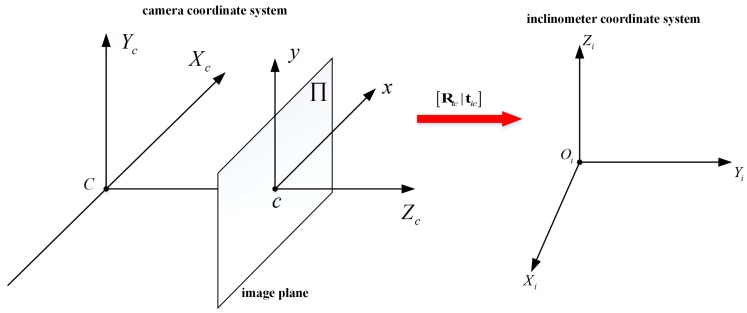
An example of the camera and inclinometer coordinate systems.

For some photoelectric measurement systems that employ inclinometers and cameras, the inclinometer coordinate system and the camera coordinate system cannot be completely overlapped during the practical installation process. Figure 1 shows an example of these two coordinate systems, where $C-X_{c}Y_{c}Z_{c}$, $O_{i}-X_{i}Y_{i}Z_{i}$ and c−xy represent the camera coordinate system, the inclinometer coordinate system, and the image plane Π, respectively. 

The transformation relationship between them can be expressed as

(1) $$X_{i}=R_{ic}X_{c}+t_{ic},$$

where $X_{c}$ and $X_{i}$ represent the coordinates of the spatial point of the camera and the inclinometer coordinate system, respectively. $R_{ic}$ and $t_{ic}$ represent the matrices of rotation and translation from the camera to the inclinometer coordinate system, respectively. 

In practice, the inclinometer is generally placed close to the camera in photoelectric measurement systems, while the distance between the measurement system and the scenario to be measured is greater than the translation distance $t_{ic}$ between the camera and the inclinometer. Thus, $t_{ic}$ can be ignored [29]. In this paper, we define the rotation matrix $R_{ic}$ from the camera coordinate system to the inclinometer coordinate system as the inclinometer assembly error, and we are aiming to calibrate this matrix by using the information in the scenario. 

## 4. Inclinometer Assembly Error Calibration and Horizontal Image Correction

**Figure 2:**
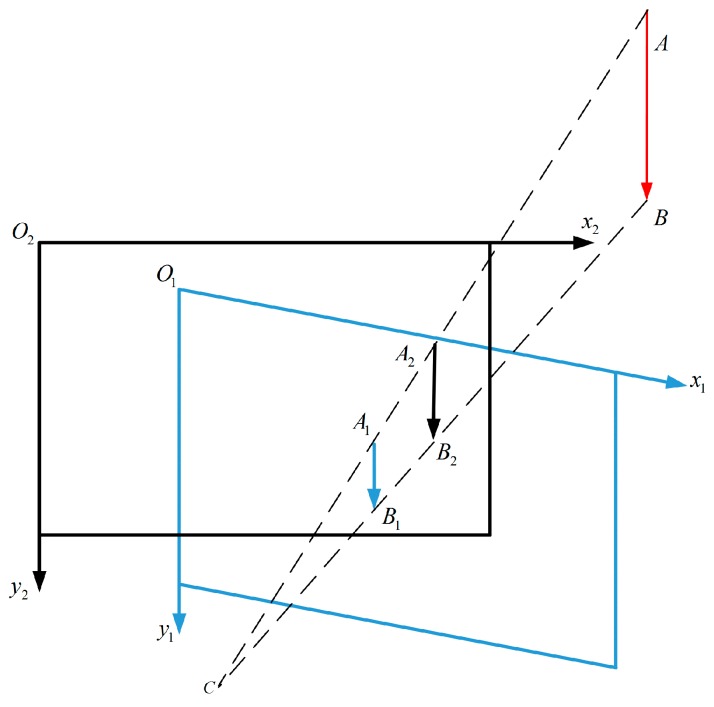
The plumb line in the real world should be a vertical line on the image plane when the camera is placed horizontally in the photoelectric system.

The inclinometer can only measure angles between two axes (x-axis and y-axis) and the horizontal plane. It cannot obtain the yaw angle value between the measurement system and the north direction. Thus, this degree of freedom should be avoided during calibration. In Figure 2, $o_{1}-x_{1}y_{1}$ and $o_{2}-x_{2}y_{2}$ represent camera coordinate systems that are in different rotation statuses, and C is the optic center of the camera. When the camera is placed horizontally, no matter how the camera’s orientation changes, the plumb line AB in the real scenario should be a vertical line $A_{1}B_{1}$ or $A_{2}B_{2}$ on the image plane, which means that points on the plumb line AB should have the same x coordinates ($x_{A_{1}}=x_{B_{1}},x_{A_{2}}=x_{B_{2}}$) in the image. Based on this principle, we propose a practical method aiming to calibrate the inclinometer assembly error $R_{ic}$, in order to avoid the limitation of the lack of the yaw angle information. 

### 4.1. Relationship between the Geodetic Coordinate System and the Inclinometer Coordinate System

**Figure 3:**
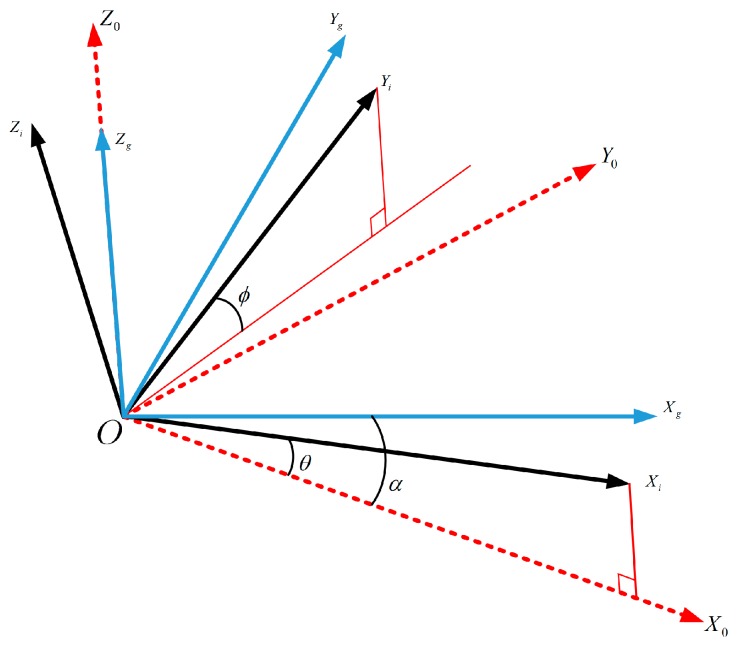
Relationship between the geodetic coordinate system and the inclinometer coordinate system.

The relationship between the geodetic coordinate system and the inclinometer coordinate system is shown in Figure 3, where $O-X_{g}Y_{g}Z_{g}$ and $O-X_{i}Y_{i}Z_{i}$ represent the geodetic coordinate system and the inclinometer coordinate system, respectively. Both of these coordinate systems follow the rules of right-handed coordinate systems. 

Rotate the geodetic coordinate system around the $Z_{g}$-axis by α degrees to make the $X_{o}$-axis the projection of the $X_{i}$-axis. Define this new coordinate system as an intermediate coordinate system $O-X_{o}Y_{o}Z_{o}$. During rotation, the inclinometer gives two angles (θ,ϕ), which are shown in Figure 3.

According to the geometric relationship, the base vector $X_{i}$ in the intermediate coordinate system $O-X_{o}Y_{o}Z_{o}$ can be expressed as

(2) $$X_{i}={\left(\cos\theta ,0,\sin\theta \right)}^{T}.$$

According to the orthogonality between the $Y_{i}$-axis and the $X_{i}$-axis, the base vector $Y_{i}$ in the intermediate coordinate system $O-X_{o}Y_{o}Z_{o}$ can be expressed as

(3) $$Y_{i}={\left(-\tan\theta \sin\phi ,\nu ,\sin\phi \right)}^{T},$$

where

(4) $$\nu =\sqrt{1-\frac{\sin^{2}\phi }{\cos^{2}\theta }}.$$

Then, the base vector $Z_{i}$ is

(5) $$Z_{i}=X_{i}\times Y_{i}={\left(-\nu \sin\theta ,-\frac{\sin\phi }{\cos\theta },\nu \cos\theta \right)}^{T}.$$

The direction cosine matrix $R_{gi}$ from the inclinometer coordinate system to the geodetic coordinate system can be represented as

(6) $$R_{gi}=R_{z}\left(\alpha \right)R_{i}\left(\theta ,\phi \right)=\left[\begin{array}{ccc} \cos\alpha & -\sin\alpha & 0\\ \sin\alpha & \cos\alpha & 0\\ 0 & 0 & 1 \end{array}\right]\left[\begin{array}{ccc} \cos\theta & -\tan\theta \sin\phi & -\nu \sin\theta \\ 0 & \nu & -\frac{\sin\phi }{\cos\theta }\\ \sin\theta & \sin\phi & \nu \cos\theta \end{array}\right],$$

where α can be interpreted as the angle between the $Y_{i}$-axis of the inclinometer coordinate system and the north direction. Changing α will not affect the value of the inclinometer.

### 4.2. Horizontal Image Correction Using the Inclinometer

**Figure 4:**
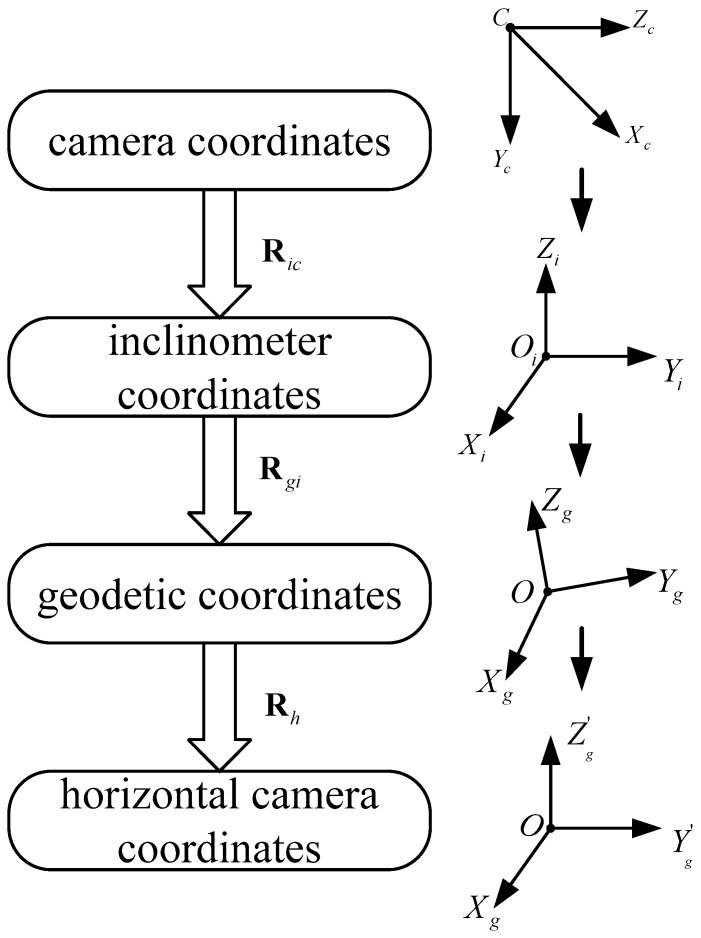
Flow chart of horizontal image correction.

Using the angle information given by the inclinometer, combined with the coordinate transformation relationship, an image taken by the camera in any attitude can be corrected to an image taken with a horizontally placed camera. Figure 4 is the flow chart depicting horizontal image correction using an inclinometer, and Algorithm 1 shows the specific steps of horizontal image correction.

**Algorithm 1:** Horizontal image correction using the inclinometer.Rotate the camera coordinate system to the inclinometer coordinate system using the rotation matrix $R_{ic}$ in Equation (1);Rotate the inclinometer coordinate system to the geodetic coordinate system according to the direction cosine matrix $R_{gi}$ in Equation (6);Rotate the geodetic coordinate system to the horizontal camera coordinate system by rotating 90° around the $X_{g}$-axis, the rotation matrix $R_{h}$ can be indicated as
(7)$$R_{h}=\left[\begin{array}{ccc} 1 & 0 & 0\\ 0 & \cos90^{\circ } & -\sin90^{\circ }\\ 0 & \sin90^{\circ } & \cos90^{\circ } \end{array}\right]=\left[\begin{array}{ccc} 1 & 0 & 0\\ 0 & 0 & -1\\ 0 & 1 & 0 \end{array}\right].$$According to homography matrix theory in multiple-view geometry [9], the homography matrix $H_{12}$ between two images can be simplified as
(8)$$H_{12}=KR_{12}K^{-1},$$
where K is the intrinsic parameter matrix of the camera, which can be expressed as
(9)$$K=\left[\begin{array}{ccc} f_{x} & 0 & c_{x}\\ 0 & f_{y} & c_{y}\\ 0 & 0 & 1 \end{array}\right],$$
where $f_{x}$ and $f_{y}$ are focal lengths in x and y directions, and $c_{x}$ and $c_{y}$ are principal points.$R_{12}$ is the rotation matrix from image 1 to image 2
(10)$$R_{12}=R_{h}R_{gi}R_{ic}=R_{h}R_{z}\left(\alpha \right)R_{i}\left(\theta ,\phi \right)R_{ic}.$$Substitute Equation (10) into Equation (8); the homography matrix $H_{12}$ can be written as
(11)$H_{12}=KR_{12}K^{-1}=KR_{h}R_{z}\left(\alpha \right)R_{i}\left(\theta ,\phi \right)R_{ic}K^{-1}.$Substitute Equations (7) and (9) into Equation (11); we have
(12)$$H_{12}=\left[\begin{array}{ccc} f_{x} & 0 & c_{x}\\ 0 & f_{y} & c_{y}\\ 0 & 0 & 1 \end{array}\right]\left[\begin{array}{ccc} 1 & 0 & 0\\ 0 & 0 & -1\\ 0 & 1 & 0 \end{array}\right]\left[\begin{array}{ccc} \cos\alpha & -\sin\alpha & 0\\ \sin\alpha & \cos\alpha & 0\\ 0 & 0 & 1 \end{array}\right]R_{i}\left(\theta ,\phi \right)R_{ic}K^{-1}.$$
(13)$$\;=\left[\begin{array}{ccc} f_{x}\cos\alpha +c_{x}\sin\alpha & -f_{x}\sin\alpha +c_{x}\cos\alpha & 0\\ c_{y}\sin\alpha & c_{y}\cos\alpha & -f_{y}\\ \sin\alpha & \cos\alpha & 0 \end{array}\right]R_{i}\left(\theta ,\phi \right)R_{ic}K^{-1}.$$Finally, the horizontal corrected image can be obtained by multiplying the homography matrix $H_{12}$ with the original image.

### 4.3. Inclinometer Assembly Error Calibration Based on Plumb Lines

**Figure 5:**
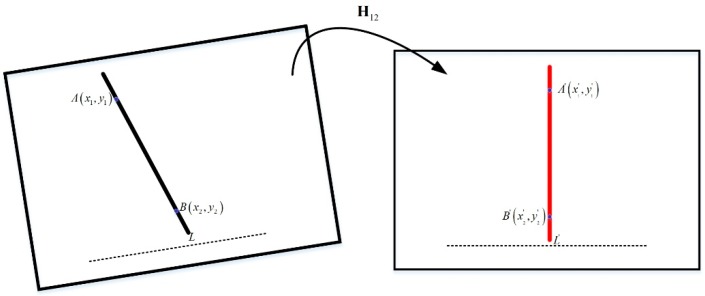
The schematic diagram of one situation.

Select two points $A\left(x_{1},y_{1}\right)$ and $B\left(x_{2},y_{2}\right)$ on the line L in the image captured by the camera in any attitude, define $A^{\prime }\left(x_{1}^{\prime },y_{1}^{\prime }\right)$ and $B^{\prime }\left(x_{2}^{\prime },y_{2}^{\prime }\right)$ as image coordinates on the vertical line $L^{\prime }$ after the homography transformation. Figure 5 shows a schematic diagram of such a situation. The plumb lines in the scenario should be vertical lines following horizontal image correction using Algorithm 1.

Based on the homography matrix theory [30], we have

(14) $$\left\{ \begin{array}{c} s_{1}\left[\begin{array}{c} x_{1}^{\prime }\\ y_{1}^{\prime }\\ 1 \end{array}\right]=H_{12}\left[\begin{array}{c} x_{1}\\ y_{1}\\ 1 \end{array}\right]\\ s_{2}\left[\begin{array}{c} x_{2}^{\prime }\\ y_{2}^{\prime }\\ 1 \end{array}\right]=H_{12}\left[\begin{array}{c} x_{2}\\ y_{2}\\ 1 \end{array}\right] \end{array}\right.,$$

where $s_{1}$ and $s_{2}$ are scale factors of homogeneous coordinates. 

Let

(15) $$\left\{ \begin{array}{c} \left[\begin{array}{c} X_{1}\\ Y_{1}\\ Z_{1} \end{array}\right]=R_{i}\left(\theta ,\phi \right)R_{ic}K^{-1}\left[\begin{array}{c} x_{1}\\ y_{1}\\ 1 \end{array}\right]\\ \left[\begin{array}{c} X_{2}\\ Y_{2}\\ Z_{2} \end{array}\right]=R_{i}\left(\theta ,\phi \right)R_{ic}K^{-1}\left[\begin{array}{c} x_{2}\\ y_{2}\\ 1 \end{array}\right] \end{array}\right..$$

Substitute Equation (13), (15) into Equation (14), and we have

(16) $$\left\{ \begin{array}{c} s_{1}\left[\begin{array}{c} x_{1}^{\prime }\\ y_{1}^{\prime }\\ 1 \end{array}\right]=\left[\begin{array}{ccc} f_{x}\cos\alpha +c_{x}\sin\alpha & -f_{x}\sin\alpha +c_{x}\cos\alpha & 0\\ c_{y}\sin\alpha & c_{y}\cos\alpha & -f_{y}\\ \sin\alpha & \cos\alpha & 0 \end{array}\right]\left[\begin{array}{c} X_{1}\\ Y_{1}\\ Z_{1} \end{array}\right]\\ s_{2}\left[\begin{array}{c} x_{2}^{\prime }\\ y_{2}^{\prime }\\ 1 \end{array}\right]=\left[\begin{array}{ccc} f_{x}\cos\alpha +c_{x}\sin\alpha & -f_{x}\sin\alpha +c_{x}\cos\alpha & 0\\ c_{y}\sin\alpha & c_{y}\cos\alpha & -f_{y}\\ \sin\alpha & \cos\alpha & 0 \end{array}\right]\left[\begin{array}{c} X_{2}\\ Y_{2}\\ Z_{2} \end{array}\right] \end{array}\right..$$

Expand Equation (16), and since $x_{1}^{\prime }=x_{2}^{\prime }$, finally we have

(17) $$X_{1}Y_{2}-X_{2}Y_{1}=0.$$

From Equation (17), we can see that angle α—defined in Equation (6)—can be eliminated during the deduction, which verifies that the changing of α will not affect the value of the inclinometer. No matter what the value α is, the plumb line will always be a vertical line following the horizontal image correction. 

In terms of actual measurement error, Equation (17) will not be strictly established. Thus, a method that minimizes the Sum of Squared Residuals (SSR) [31] is used to calculate the inclinometer assembly error $R_{ic}$. By rotating the photoelectric measurement system, multiple inclinometer values $\left(\theta_{i},\phi_{i}\right),i=1,2,\ldots ,n$ are recorded. At the same time, relative images for different inclinometer angles are captured. The Hough line-detection method [32] is applied to detect straight lines that are projections of plumb lines in the same scenario. The coordinates of the lines are recorded as $\left(x_{1}^{i},y_{1}^{i}\right),\left(x_{2}^{i},y_{2}^{i}\right),i=1,2,\ldots ,n$. The optimization function can be formed as

(18) $$\underset{R_{ic}}{\min}\frac{1}{2}\sum_{i=1}^{n}{\left(X_{1}^{i}Y_{2}^{i}-X_{2}^{i}Y_{1}^{i}\right)}^{2},s.t.\;R_{ic}^{T}R_{ic}=I,$$

where I is a 3×3 identity matrix.

In the optimization problem in Equation (18), the objective function is the Sum of Squared Residuals, and the restricted condition is the orthogonality constraint of the inclinometer assembly error matrix $R_{ic}$. Therefore, this is a nonlinear programming problem with constraints, and the matrix to be optimized, $R_{ic}$, has three degrees of freedom. In order to increase the calibration precision, it should satisfy n≥3 [33].

## 5. Experimental Results and Analyses

This section explains the experimental investigation of the inclinometer assembly error calibration in two different photoelectric measurement systems with two different cameras and inclinometers, as well as the results of the horizontal corrected image. Additionally, the calibration error is analyzed by means of both a perturbation simulation experiment [34] and a practical experiment. We also compare the proposed method with other methods.

### 5.1. Photoelectric Measurement System

The camera in the first photoelectric measurement system (defined as System 1) is a high-resolution industrial CCD VC-12MC-65 made by Vieworks Company. The lens is a Distagon 35 mm prime lens made by the Zeiss Company, which has a broad perspective, and negligible radial distortion. The camera parameters are shown in Table 1.

The dual-axis inclinometer in System 1 is made by Xi’an Sicong Chuangwei Optoelectronic Co. Ltd., which has a 0.05° angular accuracy. The inclination data acquired are all 16-bit signed numbers. The angle data in degrees can be calculated by multiplying by a scale factor of 1/3600.

**Figure 6:**
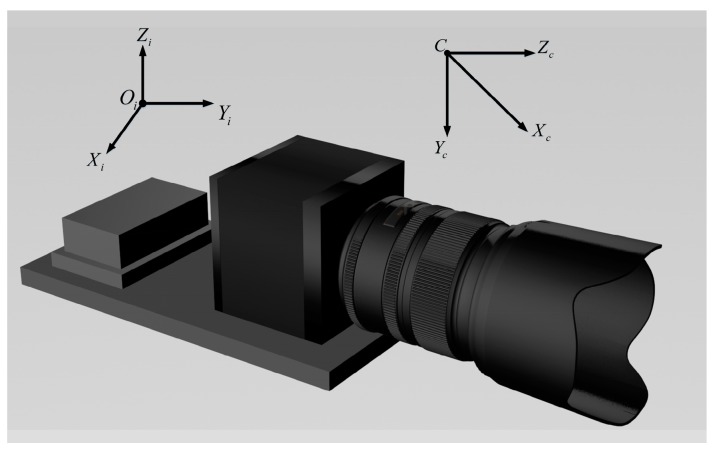
A photoelectric measurement system (System 1) with a high-resolution industrial camera and a dual-axis inclinometer.

The camera and the inclinometer are fixed on an adjustable platform. A diagram of System 1 is shown in Figure 6.

From Figure 6, we can see that the $X_{i}$-axis and the $Y_{i}$-axis of the inclinometer are orthogonal along the horizontal direction, while the $Z_{i}$-axis points to the zenith. The $X_{c}$-axis and the $Y_{c}$-axis of the camera are parallel to the x-axis and the y-axis of the image plane, respectively, while the $Z_{c}$-axis is parallel to the optic axis. Thus, a probable initial value for the nonlinear programming problem in Equation (18) for the inclinometer assembly error ${\hat{R}}_{ic}$ of System 1 could be

(19) $${\hat{R}}_{ic}=\left[\begin{array}{ccc} 1 & 0 & 0\\ 0 & 0 & 1\\ 0 & -1 & 0 \end{array}\right].$$

**Figure 7:**
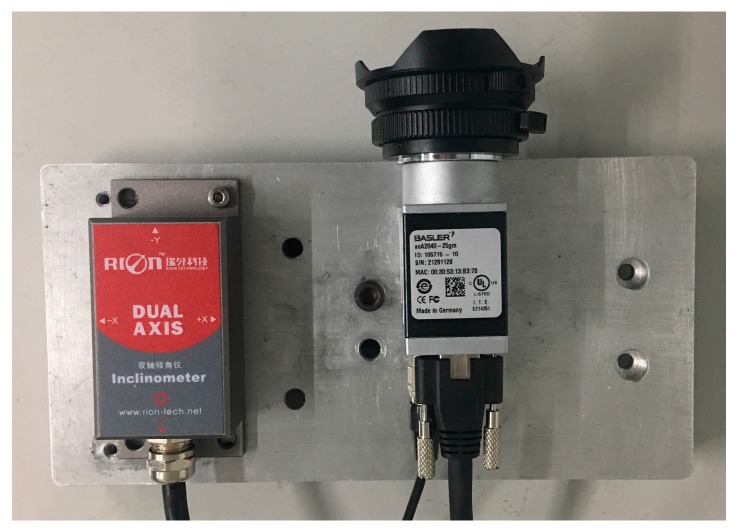
The photoelectric measurement system (System 2) by the Basler acA2040-25gm camera and the SCA126T inclinometer.

In the second photoelectric measurement system (defined as System 2), we use a Basler acA2040-25gm camera with the 8 mm fish-eye lens. This lens has significant radial distortion that needs to be corrected before the calibration. The SCA126T dual-axis inclinometer is from China Shenzhen Rion Technology Co., Ltd., with a 0.01° angular resolution. The camera parameters are shown in Table 2, and the photoelectric measurement system (System 2) is shown in Figure 7.

From Figure 7, we can also obtain one probable initial value of the nonlinear programming problem in Equation (18) for the inclinometer assembly error ${\hat{R}}_{ic}$ of System 2:

(20) $${\hat{R}}_{ic}=\left[\begin{array}{ccc} 1 & 0 & 0\\ 0 & 0 & -1\\ 0 & 1 & 0 \end{array}\right].$$

### 5.2.Calibration of Camera Intrinsic Parameter and Estimation of Lens Radial Distortion Parameter

To solve the nonlinear programming problem in Equation (18), the camera intrinsic parameter matrix K and the lens distortion parameter [35] are necessary. A commonly used lens distortion model can be written as

(21) $$\left\{ \begin{array}{l} x_{u}-x_{0}=\frac{x_{d}-x_{0}}{1+\lambda_{1}r_{d}^{2}}\\ y_{u}-y_{0}=\frac{y_{d}-y_{0}}{1+\lambda_{1}r_{d}^{2}}\\ r_{d}^{2}={\left(x_{d}-x_{0}\right)}^{2}+\alpha^{2}{\left(y_{d}-y_{0}\right)}^{2} \end{array}\right.,$$

where $r_{d}$ is the distance from the distorted point $\left(x_{d},y_{d}\right)$ to the distortion center $\left(x_{0},y_{0}\right)$, $\lambda_{1}$ is the radial distortion parameter, and α is the pixel aspect ratio. In this paper, we only consider the first term of the radial distortion, since this is enough for tasks in computer vision, according to Tsai [36].

**Figure 8:**
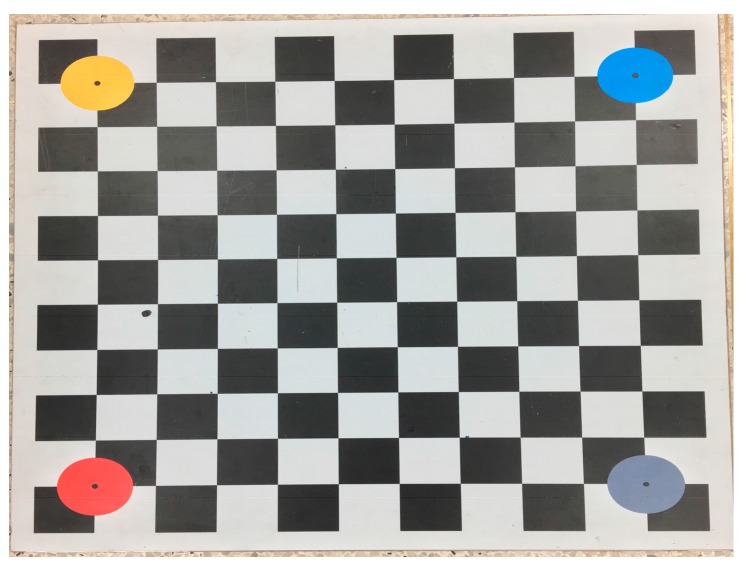
The checkerboard used for camera calibration.

We use the classical calibration method proposed by Zhang [37] to obtain the intrinsic parameter matrix and the radial distortion parameter. Figure 8 shows the checkerboard used during camera calibration. 

The intrinsic parameter matrices $K_{1}$ and $K_{2}$ of the two cameras are

(22) $$K_{1}=\left[\begin{array}{ccc} 6428.2619 & 0 & 2061.0438\\ 0 & 6425.1360 & 1546.1847\\ 0 & 0 & 1 \end{array}\right]$$

for System 1, with 0.1013 pixels mean re-projection error [34]; and

(23) $$K_{2}=\left[\begin{array}{ccc} 1343.8891 & 0 & 984.6998\\ 0 & 1345.1382 & 1051.7659\\ 0 & 0 & 1 \end{array}\right]$$

for System 2, with 0.0966 pixels mean re-projection error.

The radial distortion parameters are

(24) $$k_{1}=-0.0994$$

for System 1; and

(25) $$k_{2}=-0.3487$$

for System 2.

Images captured by System 2 will be corrected for radial distortion using the parameters in Equations (23) and (25) before Hough line detection, to reduce the coordinate detection error caused by the fish-eye lens distortion.

### 5.3. Experimental Data Measurement

During the experiment, changing the attitude of the photoelectric measurement system, taking images of each scenario $I_{i}\left(i=1,2,\ldots ,n\right)$, and recording the inclination data $\left(\theta_{i},\phi_{i}\right),i=1,2,\ldots ,n$ all takes place at the same time. When saving camera images, we make sure that there are obvious building edges in the image. Table 3a shows 12 pairs of the inclination data in degrees for System 1, and Table 3b shows the data for System 2. 

The Hough line detection method is applied to detect straight lines that are projections of plumb lines in the scenario. Other lines that are not projections of plumb lines are removed. Additionally, in order to ensure precision, only lines longer than D pixels are retained (in our experiments, D=200 pixels in System 1, and D=100 pixels in System 2). 

### 5.4. Inclinometer Assembly Error Calculation and Horizontal Image Correction

Using Equation (18), inclinometer assembly error matrices are calculated as

(26) $$R_{ic}=\left[\begin{array}{ccc} 0.9996 & -0.0014 & 0.0297\\ -0.0296 & 0.0127 & 0.9995\\ -0.0017 & -0.9999 & 0.0127 \end{array}\right].$$

for System 1; and

(27) $$R_{ic}=\left[\begin{array}{ccc} 0.9990 & 0.0197 & 0.0390\\ 0.0396 & -0.0317 & -0.9987\\ -0.0184 & 0.9993 & -0.0325 \end{array}\right].$$

for System 2.

For the original image, homography matrices $H_{12}$ are calculated with the inclination data in Table 3 by substituting Equation (22) or (23) and (26) or (27) into Equation (11):

(28) $$H_{12}=\left[\begin{array}{ccc} 0.9837 & 0.0970 & 100.0557\\ -0.1194 & 0.9824 & 521.4895\\ -0.0000 & -0.0000 & 1.0186 \end{array}\right].$$

for System 1; and

(29) $$H_{12}=\left[\begin{array}{ccc} 0.9551 & 0.1814 & -84.4734\\ -0.2094 & 0.9826 & 222.5300\\ -0.0000 & -0.0000 & 1.0274 \end{array}\right].$$

for System 2.

Finally, the horizontally corrected image is obtained by transformation using the corresponding homography matrix $H_{12}$. 

**Figure 9:**
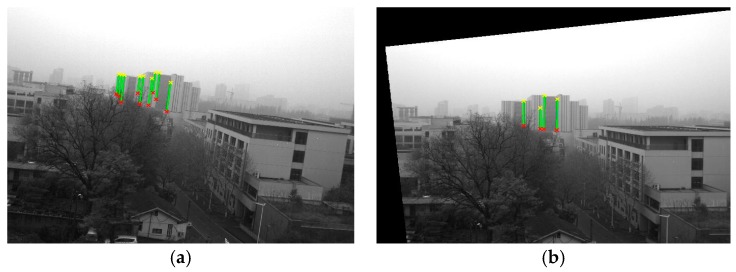
Images with detected plumb lines before and after horizontal correction in System 1. (**a**) The original image $I_{3}$; (**b**) The horizontally corrected image $I_{3\_corrected}$.

Figure 9a shows the original image, $I_{3}$, in which nine lines were detected in System 1; Figure 9b shows the horizontally corrected image, $I_{3\_corrected}$, which can be seen to be horizontally corrected, as the edges of the buildings are vertical in the corrected image. The horizontally corrected image looks better than the original one. 

**Figure 10:**
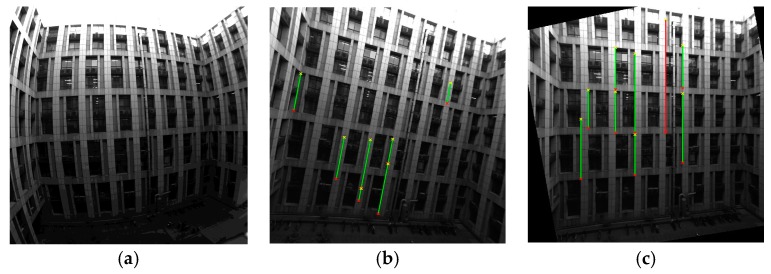
Images before and after correction using System 2. (**a**) The original distorted image, $I_{6}$; (**b**) The undistorted image, $I_{6\_undistorted}$, with detected plumb lines; (**c**) The horizontally corrected image, $I_{6\_corrected}$, with detected plumb lines.

Figure 10a–c shows the horizontal correction results of image $I_{6}$ using System 2. The original image, $I_{6}$, has serious radial distortion, which is apparent due to the plumb lines of the building having turned into curves (Figure 10a). Firstly, the image was undistorted, $I_{6\_undistorted}$ (Figure 10b), to reduce the error caused by coordinate detection, and then horizontally corrected, $I_{6\_corrected}$ (Figure 10c). 

Table 4 gives the corresponding coordinates and angle for every detected line in one image using System 2. After the horizontal correction, coordinates on the same plumb line in image $I_{6\_corrected}$ all have the same x-axis coordinate except for one, obtained by testing coordinates on the same line, and the corresponding angles with x direction are all 90°.

### 5.5. System Error Analyses

The inclinometer assembly error calibration result $R_{ic}$ in Equation (18) is deduced under ideal conditions. In real-world conditions, due to error arising during measurement and image processing, the calibration results will be affected by image noise, camera intrinsic parameter calibration errors, inclinometer angle reading errors, coordinate detection errors, etc. In order to verify the feasibility of the proposed method, one simulation experiment and one practical experiment are performed to analyze the system error. 

#### 5.5.1. Error Analyses by Simulation Experiment

In the simulated perturbation experiment, standard Gaussian random noise with a zero mean and one-pixel standard deviation is added to the endpoints of detected lines to simulate the coordinate detection error caused by image noise, the radial distortion of the lens, and the inaccuracy of the Hough line detection method.

In order to analyze the system error, the inclinometer assembly error matrix $R_{ic}$ is decomposed into Euler angles (yaw, pitch, and roll) [38]. The pseudocode of the perturbation experiment is shown in Algorithm 2.

**Algorithm 2:** Pseudocode of the perturbation simulation experiment.  For i=1:5 (i is the number of groups in the experiment)    For j=1:5 (j is the number of images taken in each group)      For k=1:10 (k is the duration over which Gaussian noise was added)        (I) Obtain image $I_{j}$, and record values of the inclinometer $\left(\theta_{j},\phi_{j}\right)$.        (II) Detect plumb lines in the image $I_{j}$ using the Hough line detection method.        (III) Add Gaussian noise to the endpoints of detected lines $\left(x_{n}^{j,k},y_{n}^{j,k}\right)$.      End    End     (IV) Calculate the inclinometer assembly error $R_{ic}^{i}$, and decompose the matrix into Euler angles.  End  (V) Calculate the average of the Euler angles as the final assembly error for each group.  (VI) Calculate the mean and the standard deviation of the Euler angle.

**Figure 11:**
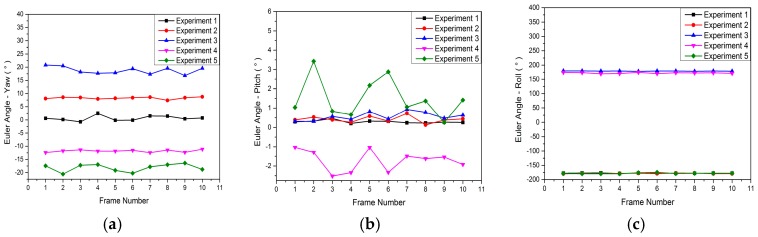
Euler angles for each experiment. (**a**) Yaw; (**b**) Pitch; (**c**) Roll.

Figure 11 shows Euler angles for each experiment. In addition, Table 5 shows the mean and the standard deviation of the Euler angles.

From Figure 11 and Table 5, we can see that the mean of the Euler angle with the Gaussian white noise illustrates the angle between the inclinometer and the camera coordinate systems, while the standard deviation with the Gaussian white noise illustrates the calibration precision, indicating the feasibility of the proposed method.

#### 5.5.2. Error Analyses by Practical Experiment

In the practical experiment, the calibration precision is tested by horizontally correcting images using the calibrated system. The pseudocode of the practical experiment is shown in Algorithm 3.

Bulleted lists look like this:
**Algorithm 3:** Pseudocode for the practical experiment.(I) Simultaneously capture the images $I_{i}\left(i=1,2,\ldots ,n\right)$ and record the corresponding inclination data $\left(\theta_{i},\phi_{i}\right),i=1,2,\ldots ,n$.(II) Detect plumb lines in the captured image $I_{i}\left(i=1,2,\ldots ,N,N=\frac{2}{3}n\right)$ using the Hough line detection method, and extract the endpoints of each line $\left(x_{i},y_{i}\right),\left(i=1,2,\ldots ,N\right)$.(III) Calculate the inclinometer assembly error $R_{ic}$ using the N images and corresponding information.(IV) Horizontally correct the n−N retained images using the calibrated matrix $R_{ic}$.(V) Detect the endpoints of the horizontal corrected images and calculate the angle with the x-axis on the same line.

**Figure 12:**
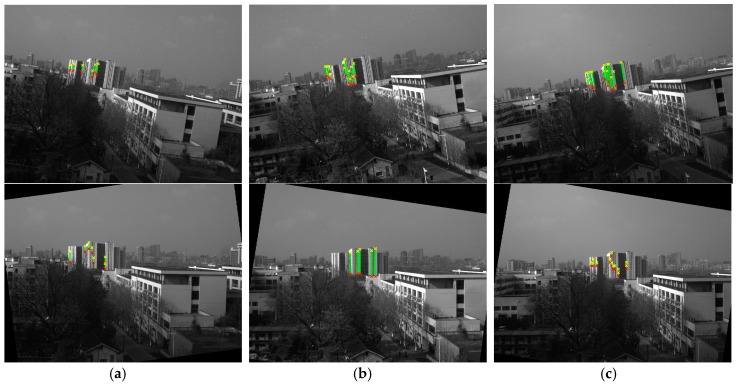
Images with lines detected before (first row) and after (second row) horizontal correction.

We first captured 9 images using the camera and inclinometer in System 1, and recorded the corresponding inclination data. Then, we used 6 of the 9 images to calibrate the inclinometer assembly error $R_{ic}$. Finally, we horizontally corrected the remaining 3 images using the calibrated $R_{ic}$. Figure 12 shows the correction results with the lines detected on them, which can be seen to be horizontally corrected, as the edges of the building are vertical in the corrected image. Table 6 provides the coordinates of the endpoints and the angle with the x direction of each line, from which we can see that most of the coordinates on the plumb line have the same x-axis coordinate after horizontal correction. It also shows that angles with the direction are 90°, which means that the calibrated result is of sufficient accuracy.

### 5.6. Comparison with Other Methods

The precision of horizontal image correction reflects the accuracy of the calibration of inclinometer assembly error in our photoelectric measurement system, since the homography matrix used for horizontal image correction is related to the inclinometer assembly error in the proposed method. In this section, we compare the proposed horizontal image correction method with three other classical image processing methods—the slant correction method based on Principal Component Analysis (PCA) [39], the image tilt correction method based on the Hough line detection method [40], and the horizontal image correction method based on the Radon method [41]—to evaluate our proposed method. 

We compare the above four methods in terms of the aspects of computation time and horizontal image correction error. In our experiment, the horizontal image correction error is defined as

(30) $$E=\frac{1}{kN}\sum_{j=1}^{k}\sum_{i=1}^{n}\left|x_{i+1}^{j}-x_{i}^{j}\right|,$$

where N is the number of plumb lines detected in the corrected image, k is the number of experiments, n=2N is the number of endpoints on the detected lines, and $x_{i+1}^{j}$ and $x_{i}^{j}$ represent the starting point and the ending point, respectively, of the same detected line. 

**Figure 13:**
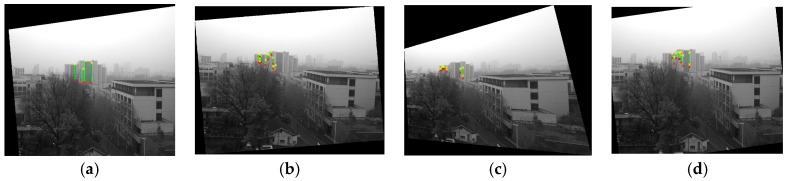
Images with lines detected after horizontal correction using the four methods. (**a**) Proposed method; (**b**) PCA method; (**c**) Hough method; (**d**) Radon method.

The comparative experiment is carried out using System 1. Figure 13 shows the horizontal correction results with lines detected using the four methods. Table 7 shows the comparison of the results, with the optimal results in boldface. 

From the comparison of the results, we can see that all the methods are able to horizontally correct the image by rotating using the inclination data, with the proposed method using the calibrated inclinometer assembly matrix to obtain the homography matrix for horizontal correction, while the other three methods use image processing methods without calibrating the inclinometer assembly error. The PCA method uses the PCA algorithm to acquire the correcting geometrical transform matrix by covariance matrix calculation and eigenvalue decomposition. The Hough method uses the horizontal lines by finding the most prominent line or edge in the image. However, it traverses the entire image for image interpolation, resulting in a longer calculation time. The Radon method is based on the rotation and projection transformation [42,43], which uses the distance from the image center pixel to the image boundary to calculate the image inclination angle.

Therefore, compared with these methods, the proposed method has the advantages of calibrating the inclinometer assembly error of photoelectric measurement systems and horizontally correcting the tilt image at the same time. The horizontal image correction error is very small compared with other methods, while the computation time is not increased, which shows the feasibility and accuracy of the proposed method.

## 6. Conclusions

This paper analyzes the expression of inclinometer assembly error in photoelectric systems. Aiming to solve the problem of the lack of yaw angle information, an inclinometer assembly error calibration method is proposed that utilizes the plumb lines in the scenario. An optimization function for calibration by deriving transformation relationships between different coordinate systems is established, and the constraint equation satisfies the inclinometer-camera system. 

The experimental results of the simulated photoelectric measurement systems show that the assembly error can be calibrated only by using the inclinometer angle information in conjunction with the plumb lines in the scenario. The perturbation experiment for the input parameter indicates the feasibility of the proposed method, which provides a theoretical basis for future work on the correction of and compensation for inclinometer assembly error, as well as improvements in accuracy and precision for photoelectric measurement systems.

Future work may include the decreasing of calibration error, and the application of the proposed method in the photoelectric detection system.

## Figures and Tables

**Table 1:** Camera parameters of the high-resolution industrial CCD in System 1.

Camera Parameter	Value
Resolution	4090 × 3072
Pixel size	5.5 μm × 5.5 μm
Device dimension	22.5 mm 16.9 mm
Focal length	35 mm
Frame frequency	64 fps
angular resolution	0.16 mrad

**Table 2:** Camera parameters of the Basler acA2040-25gm camera in System 2.

Camera Parameter	Value
Resolution	2048 × 2048
Pixel size	5.5 μm × 5.5 μm
Device dimension	11.3 mm 11.3 mm
Focal length	8 mm
Frame frequency	25 fps
angular resolution	4 MP

**Table 3:** The measured inclination data.

**(a) Inclination Data in System 1 (°)**					
**No.**	$X_{i}$**-axis Inclination**	$Y_{i}$**-axis Inclination**	**No.**	$X_{i}$**-axis Inclination**	$Y_{i}$**-axis Inclination**
**1**	1°41′21″	−0°26′12″	**7**	0°44′33″	−0°14′51″
**2**	7°15′8″	2°48′38″	**8**	−2°53′53″	−6°11′21″
**3**	−5°36′24″	−6°37′34″	**9**	−6°20′5″	−0°41′4″
**4**	1°14′16″	4°16′53″	**10**	−6°36′41″	−6°26′12″
**5**	−0°21′50″	−6°16′36″	**11**	3°42′48″	−7°30′0″
**6**	5°7′34″	−3°24′27″	**12**	3°51′33″	4°59′42″
**(b) Inclination Data in System 2 (°)**					
**No.**	$X_{i}$**-axis Inclination**	$Y_{i}$**-axis Inclination**	**No.**	$X_{i}$**-axis Inclination**	$Y_{i}$**-axis Inclination**
**1**	0.01	−0.03	**10**	−1.53	−5.75
**2**	−3.15	−1.94	**11**	−4.54	−6.57
**3**	−1.53	−1.43	**12**	6.12	−6.88
**4**	1.78	−0.20	**13**	2.18	−5.05
**5**	2.94	−0.26	**14**	−0.42	5.77
**6**	4.09	−0.59	**15**	3.05	3.25
**7**	5.75	−0.28	**16**	−2.00	4.07
**8**	7.56	−0.57	**17**	−0.04	2.95
**9**	−9.36	−1.85	**18**	0.75	0.89

**Table 4:** Coordinates and angles of each detected plumb line before and after horizontal correction.

Line No.	Before Horizontal Correction			After Horizontal Correction		
	Starting Point	End Point	Angle (°)	Starting Point	End Point	Angle (°)
**1**	(1043, 1116)	(1004, 1321)	−76.30	(1247, 334)	(1247, 518)	90
**2**	(1003, 1326)	(920, 1749)	−78.89	(1392, 133)	(1392, 239)	90
**3**	(1534, 640)	(1503, 816)	−80.01	(1115, 423)	(1115, 667)	90
**4**	(855, 1124)	(776, 1531)	−79.02	(1588, 133)	(1588, 402)	90
**5**	(775, 1536)	(755, 1639)	−79.01	(381, 485)	(382, 599)	89.50
**6**	(632, 1103)	(564, 1453)	−79.00	(303, 134)	(303, 357)	90
**7**	(263, 564)	(203, 875)	−79.08	(248, 117)	(248, 235)	90

**Table 5:** The mean and the standard deviation of the Euler angles (°).

**(a) Mean of the Euler Angles**			
**No.**	**Yaw**	**Pitch**	**Roll**
**1**	0.6415	0.2888	−178.0544
**2**	8.2894	0.4158	−178.0544
**3**	18.7872	0.5699	179.2778
**4**	−11.8215	−1.7134	172.4610
**5**	−18.1659	1.5106	−178.4386
**(b) Standard Deviation of the Euler Angles**			
**No.**	**Yaw**	**Pitch**	**Roll**
**1**	0.9574	0.0731	0.5204
**2**	0.4007	0.1694	0.8484
**3**	1.3761	0.2176	0.6053
**4**	0.4516	0.5442	1.8206
**5**	1.4514	1.0103	1.5745
AVE	0.9276	0.4029	1.0732

**Table 6:** Coordinates and angles of each detected plumb line before and after horizontal correction.

**(a) Image (a)**						
**Line No.**	**Before Horizontal Correction**			**After Horizontal Correction**		
	**Starting Point**	**End Point**	**Angle (°)**	**Starting Point**	**End Point**	**Angle (°)**
**1**	(1345, 1043)	(1327, 1169)	-81.87	(1511, 1093)	(1510, 1185)	89.38
**2**	(1564, 1031)	(1544, 1162)	-81.32	(1457, 1006)	(1457, 1120)	90
**3**	(1194, 1025)	(1178, 1137)	-80.37	(1737, 1258)	(1737, 1359)	90
**4**	(1135, 1042)	(1123, 1125)	-81.77	(1533, 1269)	(1533, 1357)	90
**5**	(1615, 1016)	(1589, 1185)	-81.25	(1735, 1374)	(1735, 1461)	90
**6**	(1519, 1134)	(1501, 1248)	-81.03	(1127, 1160)	(1127, 1348)	90
**(b) Image (b)**						
**Line No.**	**Before Horizontal Correction**			**After Horizontal Correction**		
	**Starting Point**	**End Point**	**Angle (°)**	**Starting Point**	**End Point**	**Angle (°)**
**1**	(1646, 940)	(1710, 1348)	81.09	(1827, 1250)	(1827, 1537)	90
**2**	(1630, 1004)	(1642, 1085)	81.57	(1864, 1108)	(1864, 1246)	90
**3**	(1643, 1090)	(1663, 1213)	80.76	(1864, 1252)	(1864, 1348)	90
**4**	(1336, 1063)	(1366, 1254)	81.07	(1912, 1156)	(1912, 1399)	90
**5**	(1762, 1070)	(1785, 1221)	81.34	(2056, 1319)	(2056, 1566)	90
**6**	(1787, 1232)	(1807, 1359)	81.05	(1458, 1319)	(1458, 1427)	90
**7**	(1717, 1138])	(1738, 1272)	81.09	(1801, 1258)	(1801, 1416)	90
**8**	(1739, 1277)	(1753, 1370)	81.44	(1510, 1374)	(1510, 1470)	90
**9**	(1801, 1010)	(1836, 1236)	81.19	(1771, 1307)	(1771, 1484)	90
**10**	(1369, 1058)	(1384, 1153)	81.03	(1487, 1184)	(1487, 1292)	90
**(c) Image (c)**						
**Line No.**	**Before Horizontal Correction**			**After Horizontal Correction**		
	**Starting Point**	**End Point**	**Angle (°)**	**Starting Point**	**End Point**	**Angle (°)**
**1**	(1941, 1036)	(2017, 1470)	80.07	(2284, 1350)	(2284, 1402)	90
**2**	(1966, 1032)	(2001, 1232)	80.07	(2285, 1408)	(2285, 1464)	90
**3**	(2005, 1254)	(2041, 1463)	80.23	(1998, 1296)	(1998, 1352)	90
**4**	(2173, 998)	(2235, 1353)	80.09	(2027, 1181)	(2027, 1237)	90
**5**	(1886, 1047)	(1948, 1402)	80.09	(2030, 1353)	(2030, 1409)	90
**6**	(2050, 1158)	(2092, 1400)	80.15	(2081, 1409)	(2081, 1466)	90
**7**	(1580, 1179)	(1598, 1282)	80.09	(1675, 1356)	(1675, 1412)	90
**8**	(1599, 1287)	(1620, 1407)	80.07	(1707, 1298)	(1707, 1350)	90
**9**	(1878, 1150)	(1902, 1290)	80.27	(1708, 1355)	(1708, 1412)	90
**10**	(1634, 1166)	(1675, 1401)	80.23	(1774, 1355)	(1774, 1411)	90
**11**	(1677, 1159)	(1725, 1433)	80.06	(2056, 1410)	(2056, 1466)	90
**12**	(1934, 1143)	(1967, 1331)	80.04	(2142, 1466)	(2142, 1522)	90

**Table 7:** Comparison of results of the four methods.

Method	Proposed Method	PCA Method	Hough Method	Radon Method
Computation time (s)	**7.6137**	35.3295	561.3933	9.3188
Correction error (pixel)	**0.50**	3.00	0.77	0.91
